# Development of a New Monoclonal Antibody against Brevetoxins in Oyster Samples Based on the Indirect Competitive Enzyme-Linked Immunosorbent Assay

**DOI:** 10.3390/foods10102398

**Published:** 2021-10-09

**Authors:** Xiya Zhang, Mingyue Ding, Chensi Zhang, Yexuan Mao, Youyi Wang, Peipei Li, Haiyang Jiang, Zhanhui Wang, Xuezhi Yu

**Affiliations:** 1 Henan Province Engineering Research Center for Food Safety Control of Processing and Circulation, College of Food Science and Technology, Henan Agricultural University, 63 Nongye Road, Zhengzhou 450002, China; zhangxiya@henau.edu.cn (X.Z.); dmy00117@163.com (M.D.); maoyexuan@henau.edu.cn (Y.M.); wangyouyi1213@163.com (Y.W.); 2 College of Life Sciences, Henan Agricultural University, 63 Nongye Road, Zhengzhou 450002, China; zhangchensi0719@163.com; 3 Beijing Key Laboratory of Detection Technology for Animal–Derived Food Safety, Beijing Laboratory of Food Quality and Safety, College of Veterinary Medicine, China Agricultural University, Beijing 100193, China; 18331090835@163.com (P.L.); haiyang@cau.edu.cn (H.J.); wangzhanhui@cau.edu.cn (Z.W.)

**Keywords:** brevetoxins, monoclonal antibody, enzyme-linked immunosorbent assay

## Abstract

The consumption of shellfish contaminated with brevetoxins, a family of ladder-frame polyether toxins formed during blooms of the marine dinoflagellate *Karenia brevis*, can cause neurotoxic poisoning, leading to gastroenteritis and neurotoxic effects. To rapidly monitor brevetoxin levels in oysters, we generated a broad-spectrum antibody against brevetoxin 2 (PbTx-2), 1 (PbTx-1), and 3 (PbTx-3) and developed a rapid indirect competitive enzyme-linked immunosorbent assay (icELISA). PbTx-2 was reacted with carboxymethoxylamine hemihydrochloride (CMO) to generate a PbTx-2-CMO hapten and reacted with succinic anhydride (HS) to generate the PbTx-2-HS hapten. These haptens were conjugated to keyhole limpet hemocyanin (KLH) and bovine serum albumin (BSA) to prepare immunogen and coating antigen reagents, respectively, using the active ester method. After immunization and cell fusion, a broad-spectrum monoclonal antibody (mAb) termed mAb 1D3 was prepared. The 50% inhibitory concentration (IC_50_) values of the icELISA for PbTx-2, PbTx-1, and PbTx-3 were 60.71, 52.61, and 51.83 μg/kg, respectively. Based on the broad-spectrum mAb 1D3, an icELISA was developed to determine brevetoxin levels. Using this approach, the limit of detection (LOD) for brevetoxin was 124.22 μg/kg and recoveries ranged between 89.08% and 115.00%, with a coefficient of variation below 4.25% in oyster samples. These results suggest that our icELISA is a useful tool for the rapid monitoring of brevetoxins in oyster samples.

## 1. Introduction

**Figure 1:**
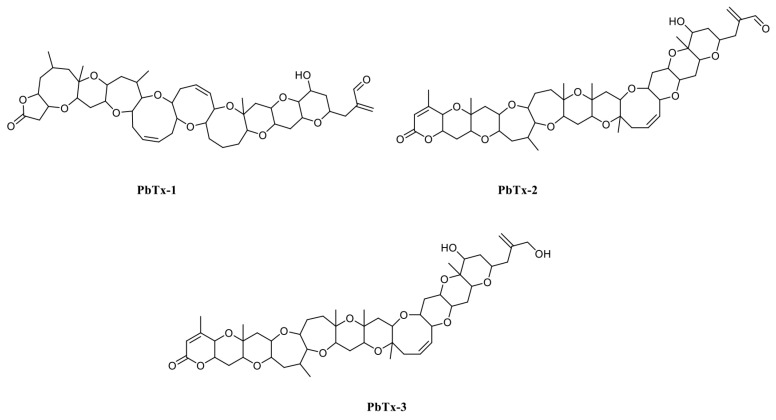
PbTx-1, -2, and -3 structures.

Marine biotoxins have negative effects on the seafood industry. Typically, they are transferred along food chains and affect other organisms, including humans. Different types of poisoning are induced by marine biotoxins, e.g., puffer fish poisoning, paralytic shellfish poisoning, scombroid fish poisoning, diarrhetic shellfish poisoning, neurotoxic shellfish poisoning, ciguatera fish poisoning, and amnesic shellfish poisoning [1]. Brevetoxins belonging to the neurotoxic shellfish poisoning group are produced by the Florida red tide organism *Karenia brevis* and are divided in two groups: (1) those derived from the brevetoxin A backbone (PbTx-1, PbTx-7, and PbTx-10) and (2) those from brevetoxin B (PbTx-2, PbTx-3, PbTx-5, PbTx-6, PbTx-9, PbTx-11, and PbTx-12) [2]. PbTx-1 is the most potent, while PbTx-2 is the most highly produced brevetoxin (Figure 1) [3]. *K. brevis* blooms occur most years in the Gulf of Mexico, killing high numbers of fish and marine mammals, including sea turtles and aquatic birds, and generate economic losses of USD 2–24 million [4]. Physiologically, brevetoxins appear to activate voltage-sensitive sodium channels causing sodium influx and nerve membrane depolarization resulting in respiratory distress [5]. Thus, to protect human health and avoid food poisoning by brevetoxins, a rapid, sensitive, and specific assay for brevetoxins detection is required.

Several analytical methods have been established to detect brevetoxins, including liquid chromatography–tandem mass spectrometry (LC-MS/MS) and receptor/antibody-based immunoassays. For example, Shin et al. (2018) developed an LC-MS/MS protocol for the PbTx-1, PbTx-2, and PbTx-3 brevetoxins with a limit of quantification (LOQ) of 25 μg/kg for each toxin [6]. Similarly, Wunschel et al. (2018) developed an electrospray LC-MS/MS system for the same brevetoxins with an LOQ of 2.5 μg/kg for each toxin [7]. Dom et al. (2018) established a high-resolution LC-MS system to detect 14 brevetoxins, with LOQs of 312 and 324 μg/kg for PbTx-2 in mussel and oyster, respectively [8]. However, these analytical methods are often time-consuming, expensive, and involve complex sample preparation. Therefore, new rapid brevetoxin screening/detection methods are required. McCall et al. (2012) developed a competitive binding assay based on rat brain synaptosomes as receptors and BODIPY^®^-PbTx-2 as competitive fluorescent probes for brevetoxin analogs [9]. In addition, Murata et al. (2019) developed a chemiluminescent receptor binding assay based on rat brain synaptosomes and acridinium-PbTx-2, with a detection limit value of 1.4 amol [10]. However, the synaptosomes were unstable and required storage at -80°C, and the assay was a time-consuming process.

Indirect competitive enzyme-linked immunosorbent assays (icELISAs) and lateral flow immunoassays (LFAs) are antibody-based and are frequently used as screening methods for small molecules due to their rapid turnaround, low costs, and high sensitivity. Recently, Zhou et al. (2010) prepared the monoclonal antibody (mAb) 2C4 using the PbTx-2-HS hapten; it exhibited IC_50_ values of 6.40, 6.57, and 5.31 μg/kg against PbTx-2, PbTx-1, and PbTx-3, respectively, with the accompanying icELISA having a limit of detection (LOD) of 0.60 ng/well [11]. Zhou et al. (2009) developed an LFA based on a colloidal gold probe for the rapid detection of brevetoxins in fish product samples, with a visual detection limit of 20 μg/kg [12]. Lai et al. (2016) developed a novel colorimetric immunoassay for PbTx-2 using an enzyme-controlled Fenton-based reaction and a 3,3’,5,5’-tetramethylbenzidine-based visual colored system, with an LOD of 0.08 ng/kg [13]. As is known, the preparation of broad-spectrum antibodies is a key step for developing an immunoassay. Hapten design is an important feature when preparing antibodies against target compounds. In the literature, PbTx-2 always reacted with succinic anhydride (HS) which introduced active sites as hapten [11,14]. In principle, aldehyde groups from PbTx-2 may be conjugated with a spacer arm, such as carboxymethoxylamine hemihydrochloride (CMO) and/or aminobenzoic acid. In addition, molecular modeling has become a powerful tool in guiding and improving hapten design strategies [15,16,17,18,19]. Therefore, we explored and developed novel haptens using molecular modeling to prepare mAbs against brevetoxins. We then developed an icELISA method to detect brevetoxins in oyster samples.

## 2. Materials and Methods

### 2.1. Materials and Reagents

PbTx-2, PbTx-1, PbTx-3, microcystins, nodularin (NOD), okadaic acid, keyhole limpet hemocyanin (KLH), bovine serum albumin (BSA), Freund’s incomplete adjuvant (FIA), Freund’s complete adjuvant (FCA), PEG1450, hypoxanthine aminopterin thymidine (HAT), and hypoxanthine thymidine (HT) were purchased from Sigma-Aldrich (St. Louis. MO, USA). Peroxidase-conjugated goat anti-mouse IgG was obtained from Jackson ImmunoResearch Laboratories, Inc. (West Grove, PA, USA). Succinic anhydride (HS), carboxymethoxylamine hemihydrochloride (CMO), N, N’-dicyclohexylcarbodiimide, N-hydroxysuccinimide, and tetramethylbenzidine were obtained from Aladdin Chemistry Co. Ltd. (Shanghai, China). Water was obtained from a Milli-Q purification system (Millipore Corp., Billerica, MA, USA). All other reagents were of analytical grade. Cell culture plates (24 and 96 wells) were from NEST (Wuxi, China). Polystyrene 96-well microtiter plates were from Costar Corp. (Cambridge, MA, USA). The absorbance at 450 nm was measured using a SpectraMax Mk3 microplate reader (Molecular Devices, Silicon Valley, CA, USA).

Coating buffer: Carbonate solution (0.05 mol/L, pH 9.60), 1.59 g Na_2_CO_3_, and 2.93 g NaHCO_3_ were dissolved in 1 L water. Assay buffer: Phosphate-buffered saline (PBS, 0.01 mol/L, pH 7.4), 8.00 g NaCl, 0.20 g KCl, 2.93 g NaHPO_4_·12H_2_O, and 0.20 g K_2_PO_4_ were dissolved in 1 L water. Washing buffer: The washing buffer comprised PBS containing 0.05% Tween 20 (PBST). Substrate solution: Solution A (pH 5) contained 1.00 g urea hydrogen peroxide, 18.00 g Na_2_HPO_4_·2H_2_O, and 10.30 g citric acid·H_2_O per liter of water. Solution B (pH 2.40) contained 0.25 g tetramethylbenzidine, 40 mL N,N-dimethylformamide, 10.30 g citric acid·H_2_O, and 960 mL water. Before the assay, solutions A and B were mixed in a 1:1 ratio [20].

Eight-week-old female BALB/c mice were provided by Vital River Laboratory Animal Technology Co. Ltd. (Beijing, China) and raised under strictly controlled conditions. The mice were manipulated according to the China Agricultural University regulations concerning the protection of animals used for scientific purposes (2010-SYXK-0037).

### 2.2. Preparation of PbTx-2-Protein Conjugates

**Figure 2:**
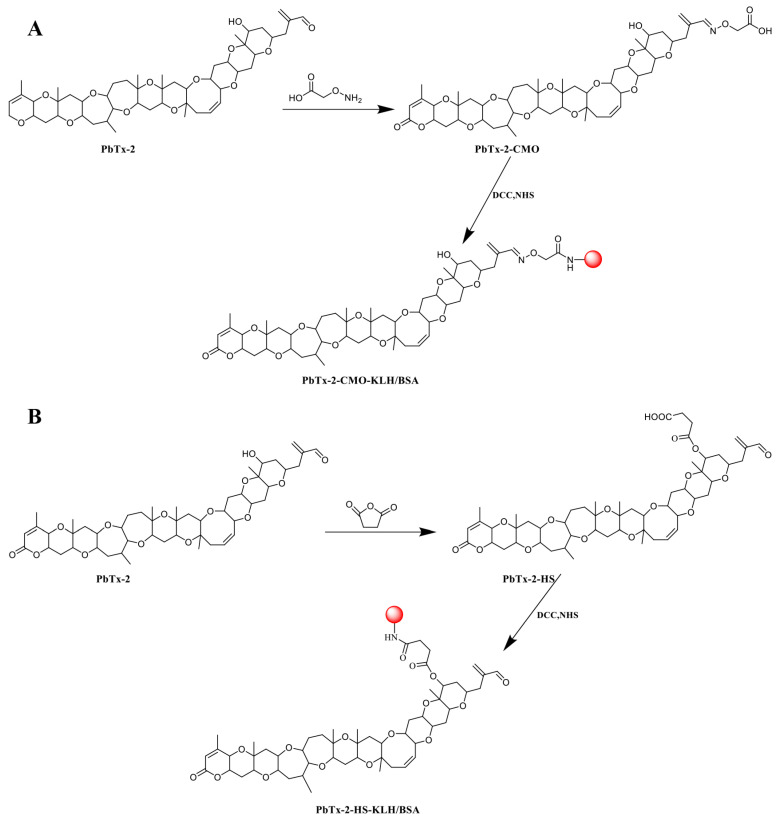
PbTx-2 hapten and antigen synthesis routes. (**A**) PbTx-2-CMO-KLH/BSA. (**B**) PbTx-2HS-KLH/BSA.

PbTx-2 was reacted with CMO to insert a carboxyl group to facilitate coupling to a carrier protein, as previously described but with modifications (Figure 2A) [21,22]. Briefly, 2 mg PbTx-2 in 5 mL pyridine was reacted with 2 mg CMO at 90°C for 6 h, after which the reaction mixture was evaporated to dryness under nitrogen gas. Then, the residue was dissolved in 5 mL 0.1 mol/L NaHCO_3_ and extracted by 5 mL ethyl acetate. The aqueous phase was adjusted to pH 3 with 0.05 mol/L HCl and extracted three times in 5 mL ethyl acetate. The organic phase was dried under N_2_ at 40 °C to generate the PbTx-2-CMO hapten. The PbTx-2-HS hapten was similarly synthesized by reacting PbTx-2 with HS as described (Figure 2B).

Then, the PbTx-2-CMO and PbTx-2-HS haptens were conjugated to KLH (immunogen) and BSA (coating antigen), respectively, via the active ester method [23]. Briefly, the haptens were respectively redissolved in 0.5 mL N,N-dimethylformamide containing 2 mg N,N’-dicyclohexylcarbodiimide and 1.5 mg N-hydroxysuccinimide and reacted for 12 h at room temperature. After centrifugation at 8000× *g* for 10 min, the supernatant of each active hapten solution was divided into two and added drop-wise to 4 mL PBS containing 5 mg KLH and 10 mL PBS containing 10 mg BSA, respectively. Reaction mixtures were stirred at 4 °C for 12 h, and the conjugates of PbTx-2-CMO-KLH/BSA and PbTx-2-HS-KLH/BSA were dialyzed in PBS for 48 h. Antigens were then stored at −20 °C.

### 2.3. Preparation of mAbs

Animal immunization procedures were as follows: twelve female BALB/c mice were immunized by subcutaneous injection. The immunogens PbTx-2-CMO-KLH and PbTx-2-HS-KLH (100 µg) were emulsified in Freund’s complete adjuvant for the first immunizations. After this, the mice were boosted with the same immunogen doses in Freund’s incomplete adjuvant every 3 weeks. Then, 7–10 days after the last immunization, serum was collected from the caudal vein. Antibody titers were analyzed by ELISA and specificity was characterized by icELISA. Animals with the highest inhibition ratios were sacrificed for fusion studies [23]. The inhibition ratio was calculated as follows:

Inhibition ratio (%) = (1 − B/B_0_) × 100%, (1)

where B_0_ and B correspond to the absorbance value of wells without a standard and the absorbance value of wells with *x* μg/kg PbTx-2 standard, respectively. Spleen cells from animals with the highest inhibition ratios were collected and fused with Sp2/0 myeloma cells using PEG1450 at a 10:1 ratio. Fusion cells were cultured in hypoxanthine aminopterin thymidine medium for 7 days. Supernatants from fusion cultures were also assayed for the titer and the inhibition ratios using ELISA and icELISA. Positive hybridomas were subcloned three times using the limiting dilution method and injected into BALB/c mice to produce ascites [23].

### 2.4. ELISA and icELISA Protocols

ELISA was conducted as previously described [24]. Briefly, (1) 100 μL of coating antigen PbTx-2-CMO-BSA (or PbTx-2-HS-BSA) diluted in coating buffer at 1 μg/mL was added to the wells of a 96-well plate and incubated at 4 °C for 10–12 h. (2) The coating antigen was then discarded, and the plate was washed three times in wash buffer (250 μL/well). (3) Then, 200 μL of 2% skimmed milk powder in assay buffer was added per well and incubated at 37 °C for 1 h to reduce unspecific binding. (4) After this, 50 μL 10 mM PBS and 50 μL anti-PbTx-2 mAb diluted in assay buffer were added sequentially to wells and incubated at 37 °C for 30 min. (5) After further washing, 100 μL/well peroxidase-conjugated goat anti-mouse IgG (diluted 1:5000) was added and incubated at 37 °C for 30 min. (6) The plate was washed five times, 100 μL/well substrate solution was added to the reaction, and the plate was incubated at 37 °C for 15 min. (7) The reaction was stopped with 50 μL 2 M H_2_SO_4_, and the absorbance was detected at 450 nm on a Multiskan MK3 microplate reader.

For the icELISA procedure, the 50 μL 10 mM PBS was replaced by 50 μL series concentration of PbTx-2 standard solution at step 4.

We characterized mAb properties using IC_50_ and cross-reactivity (CR) values. Sensitivity was assessed using IC_50_ values, with the concentration of the competitor resulting in the inhibition ratio reaching 50%, and specificity was evaluated by CR based on the following formula [24]:

CR (%) = (IC_50_ of PbTx-2/IC_50_ of competitors) × 100% (2)

We selected several marine toxin compounds as competitors, including PbTx-1 (100 μg/kg), PbTx-3 (100 μg/kg), domoic acid (1000 μg/kg), microcystins (1000 μg/kg), nodularin (1000 μg/kg), neosaxitoxin (1000 μg/kg), and tetrodotoxin (1000 μg/kg).

### 2.5. Sample Preparation

Blank oyster samples from a local supermarket were confirmed using the HPLC-MS/MS method [6]. The blank oyster samples were spiked with 200, 400, and 800 μg/kg. Then, brevetoxins were extracted from the oysters based on a previously described method with some modifications [6]. Briefly, 5 g homogenized sample was extracted in 5 mL 80% methanol by vortexing for 5 min and then ultrasonication at 60 °C for 10 min. After centrifugation at 2400× *g* for 10 min, the supernatant was diluted 15-fold in assay buffer to eliminate matrix interference. The LOD value in oyster samples was calculated based on 20 blank samples, accepting no false positive rates, with an average value plus triple standard deviation (SD), then multiplied by 15 (the diluted ratio) [23]. The recovery was calculated as the following equation [23]:

Recovery (%) = (measured value (μg/kg)/spiked values (μg/kg)) × 100%(3)

### 2.6. Molecule Alignment and Electrostatic Potential Analysis

PbTx-2, PbTx-2-CMO, and PbTx-2-HS structures were constructed using Gaussian 09 software (Gaussian Wallingford, CT, USA) according to PbTx-2 configurations in the PubChem database [15]. PbTx-2 was selected as the template to align PbTx-2-CMO and PbTx-2-HS haptens using Discovery Studio 2016 software (Accelrys Software, Inc., San Diego, CA, USA). Both Gaussian 09 and Gaussian View 5 packages were used to conduct molecular electrostatic potential (ESP) analysis.

## 3. Results and Discussion

### 3.1. Hapten Design, Synthesis, and Conjugate Preparation

Hapten design is key to generating excellent antibody performances against small molecules [25]. Generally, haptens should mimic the target molecule in terms of size, shape, electronic properties, and insert functional groups such as, carboxyl, amino, and sulfhydryl groups for carrier protein coupling [16,17]. In principle, there are two ways to synthesize PbTx-2 haptens. First, the hydroxy group of PbTx-2 is reacted with HS, which introduces active sites to construct the PbTx-2-HS hapten (Figure 2A) [11,14]. Moreover, the PbTx-2 aldehyde group is an active site which potentially reacts with an amino group with the objective of obtaining a probe or hapten. For example, McCall et al. (2012) used the active site of the aldehyde group of PbTx-2 to couple with BODIPY^®^ to synthesize the BODIPY^®^-PbTx-2 fluorescence probe [9]. Murata et al. (2019) also used this site to prepare the acridinium-PbTx-2 fluorescence probe [10]. Thus, PbTx-2 was reacted with CMO to prepare the hapten of PbTx-2-CMO (Figure 2B).

**Figure 3:**
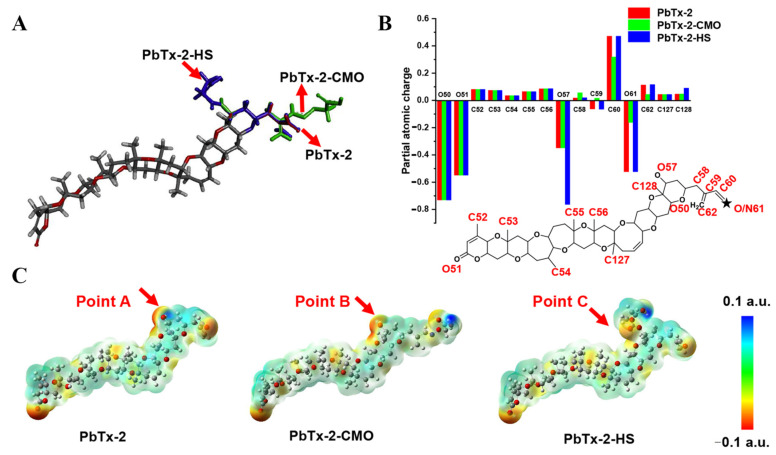
Molecular modeling results. (**A**) Overlap of PbTx-2 (gray), PbTx-2-CMO (green), and PbTx-2-HS (violet) structures. (**B**) Calculated partial atomic charges of PbTx-2, PbTx-2-CMO, and PbTx-2-HS structures. (**C**) ESP for PbTx-2, PbTx-2-CMO, and PbTx-2-HS structures. Red and blue areas indicate negative and positive potentials, respectively.

To further design the optimal hapten, PbTx-2 was selected as the template molecule to align the haptens of PbTx-2-CMO and PbTx-2-HS based on their lowest energy conformation. As shown in Figure 3A, the PbTx-2-CMO and PbTx-2-HS haptens were exposed on the left side of the structure to animal immunity. In addition, the introduction of spacer arms at the O57 or O61 position of PbTx-2 barely influenced the atom partial charges feature of PbTx-2 (Figure 3B). However, the introduction of HS hapten caused the spacer arm to form a certain angle with the parent structure of PbTx-2 (Figure 3A). To further explain the difference between PbTx-2-CMO, PbTx-2-HS, and the target compound PbTx-2 in terms of conformation and electron distribution, the ESP displayed on the van der Waals surfaces of global lowest energy conformation for PbTx-2, PbTx-2-CMO, and PbTx-2-HS is shown (Figure 3C). The PbTx-2-CMO conformation had the most similar structure to the target, PbTx-2 (Figure 3C, points A and B). The spacer HS arm of PbTx-2-HS formed a specific spatial conformation with the parent nucleus structure (Figure 3C, point C), non-conducive to the production of high-affinity antibodies against the target. Thus, PbTx-2-CMO was the ideal hapten to be used for antibody production against PbTx-2. To further verify the quality of haptens, PbTx-2-CMO and PbTx-2-HS were synthesized and conjugated with the carrier protein as complete antigens.

**Figure 4:**
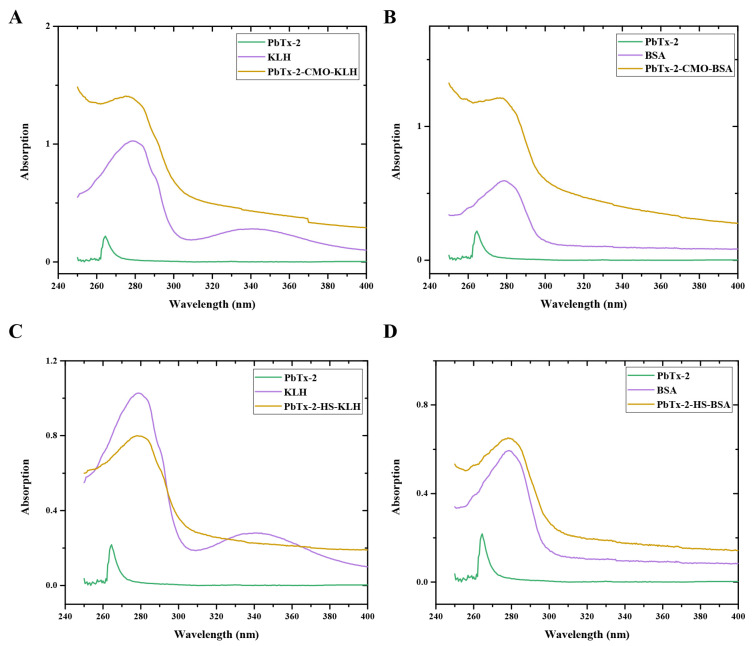
UV–visible absorption spectra of PbTx-2-CMO-KLH (**A**), PbTx-2-CMO-BSA (**B**), PbTx-2-CMO-KLH (**C**) and PbTx-2-CMO-BSA (**D**).

PbTx-2-CMO and PbTx-2-HS were activated by DCC and NHS and then conjugated with KLH and BSA as an immunogen and a coating antigen, respectively. The UV–visible absorption spectra of the immunogens, PbTx-2-CMO-KLH and PbTx-2-HS-KLH, and the coating antigens, PbTx-2-CMO-BSA and PbTx-2-HS-BSA, are shown (Figure 4). PbTx-2 had an absorbance peak at 264 nm, KLH at 280 and 350 nm, and BSA at 279 nm. The maximum absorbance peaks for PbTx-2-CMO-KLH, PbTx-2-HS-KLH, PbTx-2-CMO-BSA, and PbTx-2-HS-BSA were 275, 277, 274, and 278 nm, respectively. The maximum absorbance peaks between conjugates and PbTx-2 had shifted, thus indicating the successful synthesis of complete antigens. The calculated molar ratio of hapten to carrier protein was 1.5 for PbTx-2-CMO-KLH, 0.9 for PbTx-2-HS-KLH, 1.2 for PbTx-2-CMO-BSA, and 0.6 for PbTx-2-HS-BSA. It is accepted that the coupling ratio is an important factor affecting generated antibodies; higher coupling ratios could induce higher antibody titers [17]. In the literature, appropriate coupling ratios ranged from 3 to 15 [16,17,21]. The coupling ratios of PbTx-2-CMO-KLH/BSA and PbTx-2-HS-KLH/BSA were relatively low due to the lower reaction molar ratio (hapten to carrier protein). However, the conjugation of PbTx-2-CMO-KLH/BSA and PbTx-2-HS-KLH/BSA was successful according to the UV–visible absorption spectra (Figure 4). Therefore, these antigens were used for immunization studies.

### 3.2. Antibody Production and Characterization

The two immunogens PbTx-2-CMO-KLH and PbTx-2-HS-KLH were used to generate antibodies against PbTx-2. After a third immunization, serum was collected and characterized using ELISA and icELISA (Table 1). Mice immunized with both complete antigens produced antiserum against PbTX-2; the immune response to PbTx-2-CMO-KLH was superior to that to PbTx-2-HS-KLH from an inhibition rate perspective. However, the PbTx-2-HS-KLH antibody titer was higher than that for PbTx-2-CMO-KLH, possibly suggesting the spacer HS arm of the PbTx-2-HS molecule that formed based on molecular modeling (Figure 3A,C), a specific spatial conformation with the parent nucleus structure conduced to the production of high-titer antibodies. Additionally, all the antiserum titers from mice were low due to the lower coupling ratios of the hapten to the carrier protein. Finally, mouse No. 1 (PbTx-2-CMO-KLH) and mouse No. 5 (PbTx-2-HS-KLH) were sacrificed for fusion studies as they exhibited a higher inhibition ratio and antiserum titer.

After cell fusion, the 1D3 cell line from mouse No.1 (PbTx-2-CMO-KLH) and the cell lines 6D8 and 9E8 from mouse No. 5 (PbTx-2-HS-KLH) were identified as secreting antibodies against PbTx-2. Thus, all three cell lines were used for antibody production, and ELISAs and icELISAs were used to characterize mAbs (Table 2). All mAbs recognized PbTx-2, PbTx-1, and PbTx-3. Additionally, the IC_50_ value for the 1D3 mAb was lower than that for the 6D8 and 9G8 mAbs, consistent with the molecular modeling data (Figure 3). Those results also indicated that PbTx-2-CMO was the best hapten for brevetoxin production, and the PbTx-2-HS-BSA coating antigen improved 1D3 mAb sensitivity.

**Figure 5:**
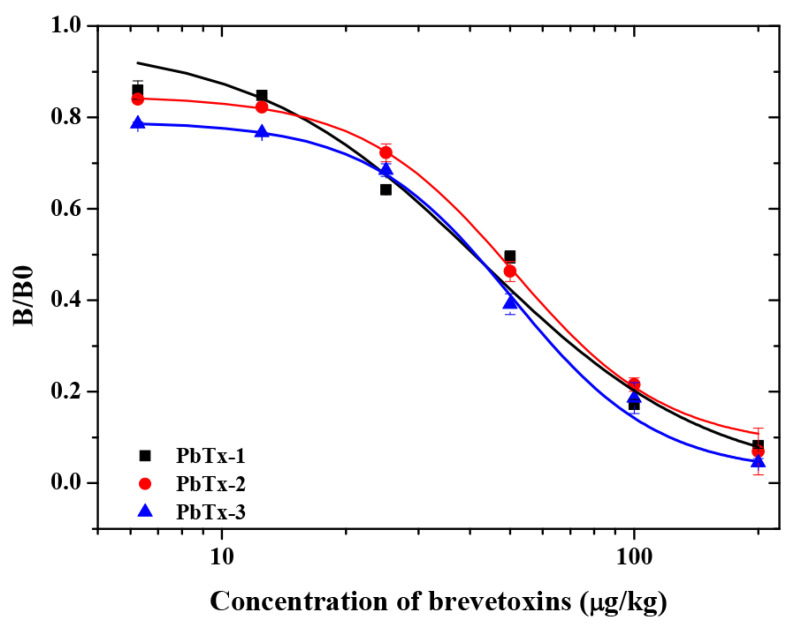
The brevetoxin icELISA standard curve using the mAb 1D3 with the PbTx-2-HS-BSA coating antigen.

Next, PbTx-2-HS-BSA and 1D3 mAb were used to establish a standard curve in buffer assay. As shown (Figure 5), the curves that were based on the PbTx-2, PbTx-1 and PbTx-3 gave IC_50_ values of 60.71 μg/kg, 52.61 μg/kg and 51.83 μg/kg, respectively. The mAb 1D3 exhibited a CR = 100% toward PbTx-2, CR = 115.40% toward PbTx-1, and CR = 117.13% toward PbTx-3. The LOD in assay buffer was 6.11 μg/kg, and the linearity range (IC_10_–IC_90_) was between 7.46 and 127.00 μg/kg. In addition, the mAb did not exhibit a measurable CR with other marine biotoxins, including domoic acid, microcystins, nodularin, neosaxitoxin, and tetrodotoxin. These data indicated that mAb 1D3 was a broad spectrum homogeneous antibody for brevetoxins.. The sensitivity of the mAb 1D3 icELISA was lower than that for the mAb 2C4 (IC_50_ value of 5.3 μg/L towards PbTx-2) [11]. In this study, the lower coupling ratios of PbTx-2-CMO to the carrier protein was because of the lower feed ratios (2 mg PbTx-2 reacted with 2 mg CMO), resulting in lower antibody titer immunized by PbTx-2-CMO-KLH. Ultimately, this affected the performance parameters of the mAb 1D3. More sensitive antibodies can be obtained if the ratios of haptens (PbTx-2-CMO) to carrier proteins are improved.

The recoveries for PbTx-2, PbTx-1, and PbTx-3 from spiked oyster samples at three dose levels (200, 400, and 800 μg/kg) are shown in Table 3 and were 91.21–108.33%, 91.04–115.00%, and 89.08–113.17%, respectively. The LOD in the oyster sample was calculated at 124.22 μg/kg. The sensitivity of this icELISA was higher than the high-resolution LC-MS sensitivity (LOQ of 324 μg/kg for PbTx-2 in oyster samples) [8] and similar to that of the LFA based on colloidal gold probe (visual detection limit of 20 μg/kg in fish product samples) by Zhou et al. [12].

## 4. Conclusions

Using molecular modeling and experimental analyses, the PbTx-2-CMO hapten produced acceptable antibody characteristics against brevetoxins. The IC_50_ values against PbTx-2, PbTx-1, and PbTx-3 were 60.71, 52.61, and 51.83 μg/kg, respectively. The LOD was 124.22 μg/kg, and PbTx recoveries from oysters ranged from 89.08% to 115.00%. This icELISA will be a useful method for monitoring PbTxs in oyster samples.

## Figures and Tables

**Table 1:** Immunization with PbTx-2-CMO-KLH and PbTx-2-HS-KLH.

Immunogen: PbTx-2-CMO-KLH/Coating Antigen PbTx-2-CMO-BSA ^a^						
No.	1	2	3	4	5	6
Absorbance of PbTx at 0 mg/kg	1.64 ± 0.06	0.52 ± 0.07	0.74 ± 0.07	0.76 ± 0.08	0.98 ± 0.07	1.542 ± 0.06
Absorbance of PbTx at 0.10 mg/kg ^c^	0.51 ± 0.02	0.15 ± 0.01	0.17 ± 0.01	0.20 ± 0.03	0.29 ± 0.05	0.63 ± 0.06
Absorbance of PbTx at 1.00 mg/kg ^c^	0.11 ± 0.02	0.07 ± 0.01	0.08 ± 0.02	0.09 ± 0.01	0.11 ± 0.01	0.14 ± 0.01
Inhibition ratio by 0.10 mg/kg	68.90%	71.15%	77.03%	84.80%	70.41%	59.14%
Inhibition ratio by 1.00 mg/kg	93.29%	86.54%	89.19%	88.16%	88.78%	90.92%
**Immunogen: PbTx-2-HS-KLH/Coating Antigen PbTx-2-HS-BSA ^b^**						
**No.**	**1**	**2**	**3**	**4**	**5**	**6**
Absorbance of PbTx at 0 mg/L	2.39 ± 0.05	0.71 ± 0.04	0.28 ± 0.01	1.07 ± 0.08	1.68 ± 0.14	0.79 ± 0.01
Absorbance of PbTx at 0.50 mg/L ^c^	2.13 ± 0.07	0.63 ± 0.05	0.17 ± 0.01	0.59 ± 0.01	1.14 ± 0.07	0.69 ± 0.04
Absorbance of PbTx at 5.00 mg/L ^c^	1.77 ± 0.03	0.58 ± 0.07	0.16 ± 0.05	0.34 ± 0.06	0.96 ± 0.06	0.49 ± 0.01
Inhibition ratio by 0.50 mg/kg	10.88%	14.86%	39.29%	44.86%	32.14%	12.66%
Inhibition ratio by 5.00 mg/kg	25.94%	18.31%	42.86%	68.22%	42.86%	37.97%

^a^ The concentration of coating antigen was 1.00 μg/mL, and the antibody titer was 1:200. ^b^ The concentration of coating antigen was 0.50 μg/mL, and the antibody titer was 1:1000. ^c^ The competition compound was PbTx-2.

**Table 2:** IC_50_ values (μg/kg) and cross-reaction (CR) of monoclonal antibodies.

Compound	PbTx-2	CR (%)	PbTx-1	CR (%)	PbTx-3	CR (%)
mAb 6D8 ^a^	786.72	100	806.52	97.92	726.51	108.29
mAb 9G8 ^a^	434.54	100	424.61	102.33	398.62	109.01
mAb 1D3 ^b^	78.53	100	80.62	97.41	69.84	112.44
mAb 1D3 ^c^	60.71	100	52.61	115.40	51.83	117.13

^a^ The concentration of coating antigen PbTx-2-HS-BSA was 0.20 μg/mL, and the antibody titer was 1:1 × 10^5^. ^b^ The concentration of coating antigen PbTx-2-CMO-BSA was 1.00 μg/mL, and the antibody titer was 1: 1 × 10^4^. ^c^ The concentration of coating antigen PbTx-2-HS-BSA was 0.20 μg/mL, and the antibody titer was 1: 3 × 10^4^.

**Table 3:** Toxin recoveries from spiked oyster samples.

Toxin	Concentration (μg/kg)	Recovery of Toxins from Oyster
PbTx-2	200	108.33 ± 3.82%
	400	91.25 ± 3.31%
	800	91.21 ± 1.25%
PbTx-1	200	115.00 ± 5.00%
	400	92.92 ± 3.82%
	800	91.04 ± 3.21%
PbTx-3	200	113.17 ± 4.25%
	400	94.42 ± 3.12%
	800	89.08 ± 1.12%

## Data Availability

Not applicable.

## References

[1] Özogul F., Hamed I. (2018). Marine-based toxins and their health risk. Food Quality: Balancing Health and Disease.

[2] Radwan F.F.Y., Wang Z.H., Ramsdell J.S. (2005). Identification of a rapid detoxification mechanism for brevetoxin in rats. Toxicol. Sci..

[3] Landsberg J.H. (2002). The effects of harmful algal blooms on aquatic organisms. Rev. Fish. Sci..

[4] Hoagland P., Jin D., Beet A., Kirkpatrick B., Reich A., Ullmann S., Fleming L.E., Kirkpatrick G. (2014). The human health effects of Florida red tide (FRT) blooms: An expanded analysis. Environ. Int..

[5] Baden D.G., Bourdelais A.J., Jacocks H., Michelliza S., Naar J. (2005). Natural and derivative brevetoxins: Historical background, multiplicity, and effects. Environ. Health Perspect..

[6] Shin C., Hwang J.Y., Yoon J.H., Kim S.H., Kang G.J. (2018). Simultaneous determination of neurotoxic shellfish toxins (brevetoxins) in commercial shellfish by liquid chromatography tandem mass spectrometry. Food Control..

[7] Wunschel D.S., Valenzuela B.R., Kaiser B.L.D., Victry K., Woodruff D. (2018). Method development for comprehensive extraction and analysis of marine toxins: Liquid-liquid extraction and tandem liquid chromatography separations coupled to electrospray tandem mass spectrometry. Talanta.

[8] Dom I., Bire R., Hort V., Lavison-Bompard G., Nicolas M., Guerin T. (2018). Extended targeted and non-targeted strategies for the analysis of marine toxins in mussels and oysters by (LC-HRMS). Toxins.

[9] McCall J.R., Jacocks H.M., Baden D.G., Bourdelais A.J. (2012). Development of a competitive fluorescence-based synaptosome binding assay for brevetoxins. Harmful Algae.

[10] Murata K., Yasumoto T. (2019). Chemiluminescent receptor binding assay for ciguatoxins and brevetoxins using acridinium brevetoxin-b2. Toxins.

[11] Zhou Y., Li Y.S., Pan F.G., Zhang Y.Y., Lu S.Y., Ren H.L., Li Z.H., Liu Z.S., Zhang J.H. (2010). Development of a new monoclonal antibody based direct competitive enzyme-linked immunosorbent assay for detection of brevetoxins in food samples. Food Chem..

[12] Zhou Y., Pan F.G., Li Y.S., Zhang Y.Y., Zhang J.H., Lu S.Y., Ren H.L., Liu Z.S. (2009). Colloidal gold probe-based immunochromatographic assay for the rapid detection of brevetoxins in fishery product samples. Biosens. Bioelectron..

[13] Lai W., Wei Q., Zhuang J., Lu M., Tang D. (2016). Fenton reaction-based colorimetric immunoassay for sensitive detection of brevetoxin B. Biosens. Bioelectron..

[14] Naar J., Branaa P., Bottein-Dechraoui M.Y., Chinain M., Pauillac S. (2001). Polyclonal and monoclonal antibodies to PbTx-2-type brevetoxins using minute amount of hapten-protein conjugates obtained in a reversed micellar medium. Toxicon.

[15] Li H., Ma S., Zhang X., Li C., Dong B., Mujtaba M.G., Wei Y., Liang X., Yu X., Wen K. (2018). Generic hapten synthesis, broad-specificity monoclonal antibodies preparation, and ultrasensitive elisa for five antibacterial synergists in chicken and milk. J. Agric. Food Chem..

[16] Mari G.M., Li H., Dong B., Yang H., Talpur A., Mi J., Guo L., Yu X., Ke Y., Han D. (2021). Hapten synthesis, monoclonal antibody production and immunoassay development for direct detection of 4-hydroxybenzehydrazide in chicken, the metabolite of nifuroxazide. Food Chem..

[17] Li Z., Wang Y., Li D., Chen X., Li Z., Gao H., Cao L., Li S., Hou Y. (2017). Development of an indirect competitive enzyme-linked immunosorbent assay for screening ethopabate residue in chicken muscle and liver. RSC Adv..

[18] Han X., Sheng F., Kong D., Wang Y., Pan Y., Chen M., Tao Y., Liu Z., Ahmed S., Yuan Z. (2019). Broad-spectrum monoclonal antibody and a sensitive multi-residue indirect competitive enzyme-linked immunosorbent assay for the antibacterial synergists in samples of animal origin. Food Chem..

[19] Huang J.X., Yao C.Y., Yang J.Y., Li Z.F., He F., Tian Y.X., Wang H., Xu Z.L., Shen Y.D. (2019). Design of novel haptens and development of monoclonal antibody-based immunoassays for the simultaneous detection of tylosin and tilmicosin in milk and water samples. Biomolecules.

[20] O’Keeffe M., Crabbe P., Salden M., Wichers J., Van Peteghem C., Kohen F., Pieraccini G., Moneti G. (2003). Preliminary evaluation of a lateral flow immunoassay device for screening urine samples for the presence of sulphamethazine. J. Immunol. Methods.

[21] Zhang X., Wen K., Wang Z., Jiang H., Beier R.C., Shen J. (2016). An ultra-sensitive monoclonal antibody-based fluorescent microsphere immunochromatographic test strip assay for detecting aflatoxin M_1_ in milk. Food Control.

[22] Zhang X., Eremin S.A., Wen K., Yu X., Li C., Ke Y., Jiang H., Shen J., Wang Z. (2017). Fluorescence polarization immunoassay based on a new monoclonal antibody for the detection of the zearalenone class of mycotoxins in maize. J. Agric. Food Chem..

[23] Zhang X., Song M., Yu X., Wang Z., Ke Y., Jiang H., Li J., Shen J., Wen K. (2017). Development of a new broad-specific monoclonal antibody with uniform affinity for aflatoxins and magnetic beads-based enzymatic immunoassay. Food Control..

[24] Peng D., Chang F., Wang Y., Chen D., Liu Z., Zhou X., Feng L., Yuan Z. (2016). Development of a sensitive monoclonal-based enzyme-linked immunosorbent assay for monitoring T-2 toxin in food and feed. Food Addit. Contam. Part A Chem. Anal. Control. Expo. Risk Assess..

[25] Wang Z., Liu M., Shi W., Li C., Zhang S., Shen J. (2015). New haptens and antibodies for ractopamine. Food Chem..

